# Visualising natural attractions within national parks: Preferences of tourists for photographs with different visual characteristics

**DOI:** 10.1371/journal.pone.0252661

**Published:** 2021-06-03

**Authors:** Lei Zhu, Lloyd S. Davis, Anna Carr

**Affiliations:** 1 Centre for Science Communication, University of Otago, Dunedin, New Zealand; 2 Department of Tourism, University of Otago, Dunedin, New Zealand; University of Florida, UNITED STATES

## Abstract

To explore what types of photographs are more helpful means to interpret natural attractions within national parks, this study focused on the relationship between the photographs with different visual characteristics and their perceived visual appeal. A photograph-based Q method was adopted. Results confirmed the visual quality of a photograph was the most important characteristic that determined its perceived attractiveness; those photographs with a high visual quality could successfully attract tourists’ attention. The subject also significantly affected the preferences of observers, suggesting an interest-dependent pattern. Using photographs of birds as examples, the participants who were interested in birds were attracted by the photographs of birds rather than those of other subjects. This study provides a better understanding of the effectiveness of photographs for communication. Findings may help researchers, communicators and national park marketers better understand and select appropriate photographs for interpretation within national parks.

## Introduction

For national parks that attract many visitors to experience nature, the interpretation of the stories about local natural attractions plays an important role for enriching the visitor experience, providing enjoyment for visitors through the propagation of the scientific stories found within the park and increasing their understanding of nature and environment [1,2].

Within the context of national parks that contain natural heritages, interpretation is defined as the communication of the facts, values and relationships of natural heritage to visitors [1]. Successful interpretation within a national park has significant implications not only for tourism but also for conservation and science communication: firstly, it helps visitors connect better with the local natural attractions and enriches their experience, encouraging repeat visiting and longer stays [3]. Secondly, it may inform visitors what the iconic nature attraction is and why it should be protected. Also, it enhances visitors’ understanding and awareness of the relevant topic (e.g. conservation, climate change, etc.), which can influence the public’s understanding and support for conservation [1,4].

When aiming to make interpretive materials more attractive and effective, existing studies and guidelines for the design of such products mainly focus on the textual and visual elements [1,5,6]. As a widely-used visual element, photographs, are commonly mentioned in interpretive materials [7], because an appropriate photograph can provide an example of a certain nature attraction and environmental/biodiversity changes within a national park [8,9].

Given the importance of photographs for interpretation, a question then raises: what types of photographs can better present local natural attractions and then attract tourists? According to the theoretical frames developed by Stuart [10] and Jenkins [11], the role and use of photographs in tourism forms a ‘circle of representation’: the photographs of local iconic attractions may be used by marketers (e.g. national parks) for interpretive and advertisement purposes. The visitors are then attracted by such photographs and decide to visit. During their visitation they may also take photographs of their preferred iconic attractions (e.g. landscapes and wildlife) and share them via social media platforms. The marketers, thereby, have a better understanding of the preferences of tourists for local attractions according to the photographs shared by the tourists. They are then able to adjust or update the photographs used for attracting more potential visitors [10,11].

The existing research on the involvement of photography in tourism within natural areas was mainly focused on tourists (i.e. the role of visitor-employed photography) rather than the use of photographs by marketers, e.g. for interpretation [12]. The photographs of natural areas that taken by visitors were proven to be closely related to visitors’ gaze, interests and local visiting experience [12,13]. Within the context of nature-based field trips, Markwell [14] also confirmed the link between photographs of local natural attractions and the preference of tourists. However, studies on the use of photographs in interpretive materials for visitors within the natural areas (rather than potential visitors) are very limited. Research on this topic concentrated more on the size, numbers and subjects of photographs used in such materials (e.g. brunches) [11,15], but the visual characteristic, particularly visual appeal of photographs is seldom considered.

Why visual appeal matters? Even though photography can play a considerable role in interpretation, not that every photograph is visually attractive to observers [16–18]. The visual characteristics of photographs, including their subjects and visual appeal, should be considered when presenting natural stories with photographs because individuals’ responses to different photographs (i.e. the perceived visual appeal) are closely related to these visual characteristics [1,17,19]. For example, Savakis, Etz [18] showed that a photograph with high visual aesthetic quality and an interesting subject is more attractive to observers. Their study showed that the subject and a few visual attributes, such as composition, lighting and sharpness are important [18]. Also, appealing images can evoke positive emotional responses that enhance attention and engagement [4,20–22]. Such positive emotional response is an important aspect of effective communication [4,23].

From the audience’s perspective, tourists themselves have their own criteria for a judging if a photograph of natural attractions within the park is appealing or not, which might be on the basis of their aesthetic preferences, their experience of visiting the park, or their interest in the subject of the photograph [24–26]. It is, therefore, important to explore tourists’ preferences for photographs with different visual characteristics in order to use appropriate photographs in interpretive materials.

However, in the context of interpreting nature within national parks, there is a lack of empirical studies that examine the attractiveness of photographs based on the influence of a combination of visual qualities and subjects. To increase the effectiveness of interpretive materials within national parks by using appropriate photographs, we aimed to clarify the preferences of tourists for the nature photographs of different subjects and the visual qualities within a Chinese national park: the Xixi National Wetland Park (XNWP) near Hangzhou City, Zhejiang Province. Given that the preferences of tourists might be affected by the visual characteristics of photographs and their interests and experiences in relation to the photographs’ subjects [27,28], the specific aims are to explore: (i) the preferences of tourists for nature photographs with different visual qualities and subjects, and (ii) whether and how their preferences are affected by the tourists’ characteristics (e.g. interests in the subject of the photograph).

## Methodology

This is a Human Subject Research. This study has been approved by the University of Otago Human Ethics Committee (ID: 17/061, written approval).

### The use of photograph-based Q method

This study focuses on the potentially shared characteristics of the preferences of tourists for photographs within a national park. The characteristics of these preferences can be extracted from participants’ explanations for the photographs they liked and disliked when given a selection of photographs to assess. A photograph-based Q method was thus adopted for this study. This method is a widely applied approach to correlating respondents’ subjective perceptions or preferences for a selection of photographs, then generating those shared patterns through factor analysis, so that participants’ preferences can be described and interpreted through a few factors [29,30].

The procedure for conducting Q method interviews is generally similar across different studies. To conduct a photograph-based Q method interview, respondents need to sort photographs based on their preferences and give an explanation for the result (e.g. why they like or dislike a photograph). Explanations from respondents are important when interpreting the result because they can reflect respondents’ underlying attitudes and can potentially reveal the link between the preferences of participants and the characteristics of photographs [24,30,31]. A study using Q method generally does not require a large sample size because the explanations from respondents can be used to complement the results when conducting a survey with a relatively small sample population [24,32,33]. Furthermore, the subsequent factor analysis is an indispensable part of the method. However, in contrast of the traditional factor analysis that explores the correlations between variables within samples, the Q method uses an inverted factor analysis process, which looks at the potential commonality in subjects across a number of variables [34]. The subjects (individuals’ evaluations and viewpoints) in the Q method are, therefore, treated as the variables in traditional factor analysis. Through interpreting the factors extracted, different patterns of preferences can be defined and described [25,27,30].

The use of a photograph-based Q method was appropriate here because it links different types of photographs to the visitors’ preferences and interests. Specifically, a selection of photographs (defined as the Q set), reflecting a variety of natural attractions within XNWP, were provided to participants. Participants were able to sort these photographs based on their experiences, personal preferences and interests. The results of sorting are called the Q sorts [31]. The degree of similarities of the sorting between participants potentially helps to identify the subjects and visual qualities of photographs that are best for interpretation when using in the park.

### The design of the experiment

A total of thirty photographs were selected as the Q set (Table 1). The visual qualities of the selected photographs in the Q set were measured automatically by an online approach: Acquine. This webpage-based evaluation system uses computational models to extract and assess the aesthetic values of the uploaded photographs [35]. The selected photographs were diverse [24,25], covering a wide range of visual aesthetic qualities (with scores by Acquine ranging from 2.2 to 10.0) and a wide range of natural science attractions within XNWP. Some of these photographs were taken by the author while others were drawn from the internet. All the photographs downloaded from the internet were approved to use in this project under the Creative Common License [36].

**Table 1:** The Q set (thirty photographs in total).

Category	The subject of the selected photograph	Field of view	Photo ID	Aesthetics
Local landscape	Pond with wetland vegetation	wide view	WV01	8.4
	Pond with wetland vegetation	medium view	WV04	5.8
	Pond with wetland vegetation	wide view	WV03	3.3
	Wetland, forests and bridge	medium view	RT02	5.1
	Wetland, reed and bridge	wide view	RT01	4.6
Local birds	Small wetland bird—Common Kingfisher	close-up view	CK03	8.9
	Small wetland bird—Common Kingfisher	close-up view	CK01	7.5
	Small wetland bird—Common Kingfisher	close-up view	CK06	4.6
	Intermediate wetland bird–Mandarin Duck	close-up view	MD05	8.7
	Intermediate wetland bird–Mandarin Duck	close-up view	MD01	5.4
	Intermediate wetland bird–Mandarin Duck	close-up view	MD03	7.7
	Large wetland bird—Little Egret	close-up view	LE01	8.1
	Large wetland bird—Little Egret	close-up view	LE06	8.5
	Large wetland bird—Little Egret	close-up view	LE02	5.9
	Small passerine–Vinous-throated Parrotbill	close-up view	VP03	8.5
	Small passerine–Vinous-throated Parrotbill	close-up view	VP01	7.7
	Small passerine–Vinous-throated Parrotbill	close-up view	VP02	6.2
	Intermediate passerine–Light-vented Bulbul	close-up view	LB01	8.2
	Intermediate passerine–Light-vented Bulbul	close-up view	LB04	10.0
	Intermediate passerine–Light-vented Bulbul	close-up view	LB06	6.2
	Large passerine—Red-billed Blue Magpie	close-up view	RM06	7.4
	Large passerine—Red-billed Blue Magpie	close-up view	RM02	5.7
	Large passerine—Red-billed Blue Magpie	close-up view	RM04	2.2
Wildlife other than birds	Black-spotted Frog	close-up view	RG01	8.6
	Globe Skimmer Dragonfly	close-up view	PZ01	10.0
Local plants	Forest	wide view	FR01	6.7
	One tree in the forest	close-up view	FR02	5.8
	Shrub	medium view	BS01	7.1
Local facilities	A bird-watching hide	close-up view	HD01	6.1
	An interpretive sign (bird topic)	medium view	SN01	5.9

It includes what natural attractions were presented and how they were presented (field of view). The aesthetic scores were given by Acquine. The Common Kingfisher (*Alcedo atthis*), Mandarin Duck (*Aix galericulata*), Little Egret (*Egretta garzetta*), Vinous-throated Parrotbill (*Sinosuthora webbiana*), Light-vented Bulbul (*Pycnonotus sinensis*), Red-billed Blue Magpie (*Urocissa erythroryncha*), Black-spotted Frog (*Pelophylax nigromaculata*) and Globe Skimmer Dragonfly (*Pantala flavescens*) are locally common species.

As the main attractions in this national park are the wetland landscape, plants and wildlife (mainly birds), the photographs in the Q set included these subjects, plus a few local facilities in relation to the nature tour. All the species in the photographs are common within XNWP. Birds were the subject of eighteen out of the total thirty photographs. These eighteen photographs covered six species of local birds, including both wetland birds and forest/shrub birds [37]. The reason for using different species of birds is that their morphological traits, taxa and habitats may affect the observers’ preferences for photographs of this type of bird [28,38–40]. Each species of bird had three different photographs with different visual qualities, including at least one photograph with high visual quality (scored above 7.0) and at least one poor-quality photograph (scored below 7.0) [35], see Table 1.

### Definition of participants’ interests in birds

The photographs of birds comprised 60% of the Q set (eighteen out of thirty) with different species and a range of aesthetic values because birds are one of the major natural attractions within XNWP [41,42]. To avoid potential bias from personal interests when testing the attractiveness of the photographs [39], participants’ interests in birds were examined and grouped [4]. Participants were divided into three interest groups during the Q method interview by self-evaluation: (i) specialised bird enthusiasts—people with a specialised interest in birds (SB), (ii) people with a general interest in birds (GB) and (iii) those are not interested in birds (NB). Specifically, SB do serious bird watching, or their career (e.g. job or study) is directly related to birds. Thus, they have good knowledge of birds. GB are interested in birds generally but do not have much experience and knowledge of birds. Those identified as NB professed not to care about birds much if at all. The above interests of the participants were used to describe the characteristics of participants loaded on each factor extracted through the Q method.

### Interview procedures

All the participants were over eighteen years of age. Instead of random sampling, participants need to be diverse for Q methods, and the focused characteristics (i.e. interest in birds in this project) should be balanced [32]. Specifically, the proportion of the three interest groups (SB, GB and NB) should be approximately equal. In order to meet these requirements, some of the participants were recruited within XNWP (i.e. tourists in the park, mainly NB or GB) by personal invitation. Other participants (mainly SB) were recruited with the help from the local birdwatching organisation: Zhejiang Wild Bird Society. Through the approaches above, those individuals who had visited XNWP recently (within six months) were encouraged to participate in the interview.

**Fig 1:**
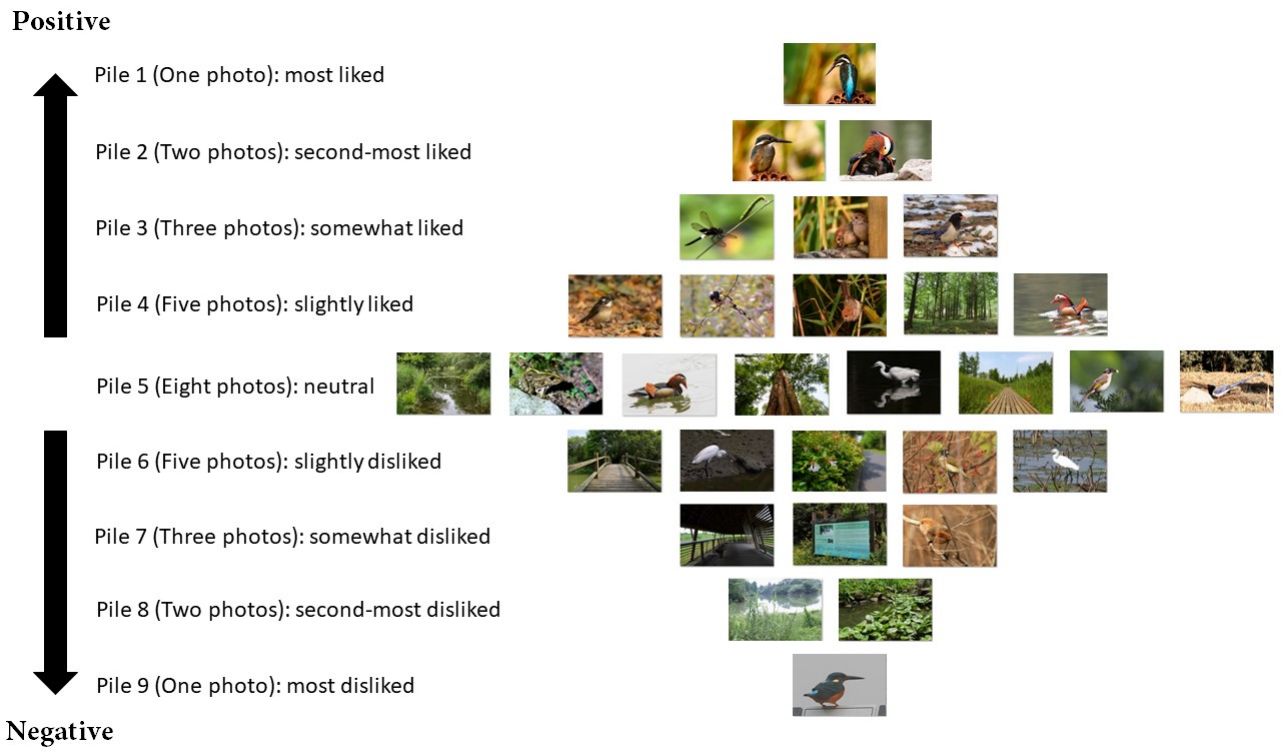
The structure of a Q sort. An example of how the photographs were grouped into the nine piles by a certain participant (Q sorting). Piles represented different attitudes towards the photographs. The completed Q sort was, therefore, a diamond shape, reflecting a template of a normal distribution. Due to copyright restrictions, some photographs here figure are similar but not identical to the original photographs used in the Q set and is therefore for illustrative purposes only. All the photographs included in this figure are taken by the author (Lei Zhu).

Interviews were conducted from May 2017 to July 2017. Each interview involved one interviewer and one participant and followed a set procedure. The interviewer introduced himself as the start of an interview, then briefly described this project as well as the procedure for the interview. An information sheet and a consent form were then provided. The participant would then ensure that he/she had read the information sheet and signed the consent form. Next, the interviewer noted a few characteristics of the participant (gender and interest in birds) using a smartphone. The next step was sorting the photographs, which was the vital part of the interview, following the protocol of a typical photograph-based Q method survey [25,43]: The participant was given the thirty photographs (i.e. the Q set) and was asked to sort all the photographs into nine piles according to the question: *For these photographs that show natural attractions of XNWP*, *what photographs do you like or dislike*? The nine piles thus represented the participants’ different evaluations of these photographs. The set of piles and the quantities of photographs to be placed in each pile briefly resembled a normal distribution of liked photos, neutral and disliked photos [24,30,43]. For details see Fig 1 as an example of a completed Q sort. The participant was then asked to explain the reasons for choosing the three most and second-most liked and disliked photographs. During the interview, we did not provide the participants with any guidelines (criteria) to help them explain why they liked or disliked a certain photograph, meaning that participants had to explain their preferences based on their own perceptions. As a participant only produced one Q sort, the total number of Q sorts was equal to the sample population of the survey.

### Q analysis and interpretation

All the Q sorts produced by the participants were processed as follows to undertake the subsequent factor analysis. As shown in Table 2, different scores were assigned to the nine piles within the Q sorts, from -4 (the most disliked pile of photographs) to +4 (the most liked piles of photographs), meaning that each photograph had a score given by each participant [24]. The factor analysis showed the potential correlations between different Q sorts. A few factors were then extracted and interpreted. The software used for this part of the process was PQMethod (Version 2.35), which was frequently used for Q method analysis [29].

**Table 2:** The Q sort distribution is designed briefly based on a normal distribution.

Number of photos per pile	1	2	3	5	8	5	3	2	1
Score of each pile	-4	-3	-2	-1	0	+1	+2	+3	+4

Qualitative content analysis of participants’ explanations for their choices was also applied to help interpret each factor, because these explanations not only reflected the participants’ perceptions of their liked or disliked photographs but also presented the potential link between their preferences and the characteristics of photographs (e.g. subjects and aesthetics) [32,33].

## Results

### Factors extracted and interpretations

A total of thirty-six participants, covering an approximately equal number of SB (twelve), GB (thirteen) and NB (eleven) participated in the interviews. All thirty-six completed Q sorts were photographed and then inputted into the database. Explanations for the Q sorts by participants were recorded in audio or in text, depending on the choice of the participant.

Four factors were extracted via the factor analysis after a varimax rotation [29], explaining 71% of the total variance. Results showed the participants’ varied and distinctive preferences through four factors, which were likely to be influenced by personal interests, appreciation and experiences. Thirty-three out of the thirty-six participants were significantly loaded on one of the four extracted factors and were used to describe each factor, reflecting that these participants had shared characteristics of their preferences for photographs (NB: significant loadings were detected and marked by the software). The remaining three participants did not have a significant loading or had multiple loadings. The Q sorts produced by these three participants could not be used to define any single factors and were, therefore, excluded from the factor analysis [29,44]. The scores given by each participant (i.e. original data set) were reported in S1 Table.

#### Factor 1: Wildlife photographs with outstanding aesthetic value

This factor is defined by the Q sorts of thirteen participants (i.e. significant loadings), including seven GB, five NB and only one SB. It comprises 27% of the total variance. The six photographs with the highest and lowest Q-sort scores were listed in Table 3.

**Table 3:** The six top-ranked and six bottom-ranked photographs for Factor 1.

	ID	Subject	Acquine score	Q score
Top-ranked Photos	MD05	Mandarin Duck	8.7	+4
	CK03	Common Kingfisher	8.9	+3
	CK01	Common Kingfisher	7.5	+3
	RM06	Red-billed Blue Magpie	7.4	+2
	LB04	Light-vented Bulbul	10.0	+2
	PZ01	Globe Skimmer Dragonfly	10.0	+2
Bottom-ranked Photos	RM04	Red-billed Blue Magpie	2.2	-2
	SN01	An interpretive sign	5.9	-2
	WV03	Wetland vegetation	3.3	-2
	CK06	Common Kingfisher	4.6	-3
	LB06	Light-vented Bulbul	6.2	-3
	VP02	Vinous-throated Parrotbill	6.2	-4

Not all the participants engage with birds (see the interest groups), but they do like appealing photographs: the visual aesthetic qualities of photographs became the most important factor amongst all four factors. All the six photographs are of high visual quality (Acquine score over 7.0), including the first, second, third and fourth highest scoring photographs in the Q set: PZ01 (10.0), LB04 (10.0), CK03 (8.9) and MD05 (8.7). The subjects of these six photographs are all wildlife: local wetland birds (the Mandarin Duck and the Common Kingfisher) and local forest/shrub birds (the Red-billed Blue Magpie and the Light-vented bulbul), as well as a wetland insect species (the Globe Skimmer Dragonfly). According to the statements from participants, high-ranking photographs for this factor were mainly described as clear, sharp, colourful and full of actions.

On the other hand, the bottom six photographs cover a variety of subjects, including local wildlife, vegetation and tourism facilities. However, it is important that all of them are poor-quality photographs. Here, negative statements mainly focused on blur and colourless subjects, not beautiful, not attractive, background too complicated, and so forth.

#### Factor 2: Local birds encounter

A total of seven participants were significantly loaded here, including five SB, two GB and no NB. Factor 2 accounts for 16% of the total variance. Compared to Factor 1, which reflected aesthetic-dependent preferences, Factor 2 showed a clear pattern of subject-related preferences. Participants associated with this factor focused specifically on birds. This factor was named as *Local Birds Encounter*. Table 4 presents the six photographs with the highest Q scores and the six with the lowest Q scores.

**Table 4:** The six top-ranked photographs (a) and the six bottom-ranked photographs (b) for Factor 2.

	ID	Subject	Acquine score	Q score
Top-ranked Photos	RM06	Red-billed Blue Magpie	7.4	4
	CK03	Common Kingfisher	8.9	3
	RM02	Red-billed Blue Magpie	5.7	3
	LB04	Light-vented Bulbul	10.0	2
	LE01	Little egret	8.1	2
	VP01	Vinous-throated parrotbill	7.7	2
Bottom-ranked Photos	RT02	Forest and bridge	5.1	-2
	HD01	A birdwatching hide	6.1	-2
	WV03	Wetland vegetation	3.3	-2
	WV04	Wetland vegetation	5.8	-3
	CK06	Common Kingfisher	4.6	-3
	SN01	An interpretive sign	5.9	-4

All the participants loaded on this factor are interested in birds (i.e. SB and GB, with no NB). These participants, especially the bird enthusiasts, knew and had encountered many species of birds in the wild. These knowledgeable participants tended to evaluate the subject from a bird watcher’s point of view: whether they were impressed by the behaviour and ecology of the bird in the photograph, and whether they thought the bird was rare or representative within XNWP. For example, one of their reasons for choosing the Common Kingfisher (CK03) was that it appeared with a fish, which reflects both its habitat (wetland) and its typical behaviour. By contrast, GB, from their comment, did not care much about habitat and ecology. Instead, they seemed to be attracted by some morphological traits of the birds. A participant who was loaded on this factor and had a general interest in birds, for example, preferred the photographs of a Red-billed Blue Magpie simply because it has an amazingly long tail. Also, for some locally common species, those GB would vote for them if they had seen them within the park.

#### Factor 3: Iconic landscape and environment within XNWP

Factor 3 had nine participants (9 Q sorts) loaded, explaining 18% of the overall variance. Amongst the participants that defined Factor 3, four were SB, four were NB, and one was GB. Highly commended photographs for this factor were those of local iconic landscapes and vegetation. For example, three of the photographs showed different types of local landscapes: FR01 for the forest, WV01 for the wetland (river and vegetation nearby), and BS01 for shrub vegetation along the walking track. Especially, VP01, as the most preferred photograph by participants for this factor, presented a typical wetland path surrounded by reed (background). Accordingly, this factor was named as *Iconic Landscape and environment within XNWP*. Six photographs with the highest and the lowest scores are listed in Table 5. Even though these SB and NB evaluated photographs from different perspectives, they still reached consensus. They liked the natural and locally representative wetland environment. As a result, their choices of photographs mainly included those that contained or reflected this type of environment.

**Table 5:** The six top-ranked photographs (a) and the six bottom-ranked photographs (b) for Factor 3.

	ID	Subject	Acquine score	Q score
Top-ranked Photos	VP01	Vinous-throated Parrotbill	7.7	4
	CK03	Common Kingfisher	8.9	3
	CK01	Common Kingfisher	7.5	3
	FR01	Forest	6.7	2
	WV01	Wetland vegetation	8.4	2
	BS01	Shrub	7.1	2
Bottom-ranked Photos	RM04	Red-billed Blue Magpie	2.2	-2
	LB06	Light-vented Bulbul	6.2	-2
	WV04	Wetland vegetation	5.8	-2
	LE02	Little Egret	5.9	-3
	WV03	Wetland vegetation	3.3	-3
	CK06	Common Kingfisher	4.6	-4

#### Factor 4: Wetland plants and animals within XNWP

Four participants significantly loaded here, including three GB and one NB, but no SB. This factor only accounts for 10% of the total variance, but it still reflects the interest of participants in wetland plants and wildlife. Six photographs with the highest and lowest scores are listed in Table 6.

**Table 6:** The six top-ranked photographs (a) and the six bottom-ranked photographs (b) for Factor 4.

	ID	Subject	Acquine score	Q score
Top-ranked Photos	VP01	Vinous-throated Parrotbill	7.7	4
	CK01	Common Kingfisher	7.5	3
	PZ01	Globe Skimmer Dragonfly	10.0	3
	CK03	Common Kingfisher	8.9	2
	WV01	Wetland vegetation	8.4	2
	RM02	Red-billed Blue Magpie	5.7	2
Bottom-ranked Photos	BS01	Shrub (close-up shot)	7.1	-2
	LE06	Little egret	8.5	-2
	WV03	Wetland vegetation	3.3	-2
	HD01	A birdwatching hide	6.1	-3
	SN01	An interpretive sign	5.9	-3
	CK06	Common Kingfisher	4.6	-4

### Implications of interests in birds

As the subjects of the majority of photographs used in this study were local birds, participants’ interests in birds were taken into account to describe the characteristics of participants (defination of different interests see methodology). With regard to knowledge, SB, as bird enthusiasts or specialised bird watchers, are knowledgeable about birds, while GB and NB are generally not as knowledgable. As to interests, both SB and GB are interested in birds. By contrast, NB are not interested in birds. Table 7 shows how different sets of people (based on the three interests above) responded to the photographs.

**Table 7:** The characteristics/preferences of SB, GB and NB.

Interest	Factor loading	Description
SB	F2 (5) > F3 (4) > F1 (1) > F4 (0)	Their preferences are closely related to birds, including species, behaviour and ecology presented in the photo. Their local birding experience also plays a role.
GB	F1 (7) > F4 (3) > F2 (2) > F3 (1)	They prefer aesthetically appealing photos, especially those that reflect the iconic local environment and wildlife (i.e. wetland and wetland wildlife, especially birds).
NB	F1 (5) > F3 (4) > F4 (1) > F2 (0)	They are attracted by aesthetics and enjoy a wide range of local landscapes and environment.

F1 = Factor 1, F2 = Factor 2, F3 = Factor 3, F4 = Factor 4. Factors were sorted based on the numbers of participants loaded. For each interest group, the numbers in brackets present the number of participants significantly loaded on different factors.

As presented in Table 7, the preferences of participants with different interests in birds are distinctive. Specifically, when evaluating the attractiveness of a given photograph, bird enthusiasts (SB) apparently prefer photographs of birds (i.e. the subject) rather than the aesthetics of a photograph. By contrast, the visual quality factor (Factor 1) is more important for GB and NB. Moreover, GB preferred photographs of the iconic environment and wildlife (related to the theme of the national park, i.e. wetland), while those NB could be attracted by a variety of types of subjects including landscape, vegetation and wildlife. The results above show that people’s interests in the primary subject (taxa) of the photo were indeed taken into account when they evaluate whether a photograph is appealing.

## Discussion

### The role of visual aesthetics

As shown in the results, the visual quality of a photograph is indeed one of the most important factors that determine its perceived attractiveness. The factor that represents the influence of visual qualities (i.e. Factor 1) on participants’ preferences explained the largest proportion (27%) of the total variance. While the visual qualities were manipulated to include a range from low to high, this finding, nevertheless, suggests that tourists are able to discern the aesthetic value of a photograph. Specifically, when choosing preferred photographs amongst a selection with a similar subject but different visual qualities, participants showed great interest in the high-quality photographs and avoided the low-quality ones, especially for the thirteen participants loaded on Factor 1.

Individuals’ aesthetic appreciation of photographs is a complex and highly subjective topic: everyone has his/her own personal taste when judging the visual quality of a photograph. To assess the quality of photographs in an objective way, looking for potential consensus of aesthetic preferences has become a widely-discussed topic [45–47]. The present study shows that consensus of visual aesthetic appreciation, which was reflected in participants’ preferences for photographs, indeed exists: photographs with high aesthetic scores could successfully gain more attention. Similarly, Husain, Roy [48] reviewed different types of nature and wildlife photography and concluded that photographs with high aesthetic appeal could get people’s attention and enhance conservation. This study provided empirical evidence for the link between the visual aesthetic quality of photographs and participants’ attention (reflected by preferences).

In summary, outstanding nature photographs can successfully get the participants’ attention. Similarly, a few other studies also showed the visual quality of photographs significantly attract observers [17,49]. A possible explanation is that observers have some common preferences for a few visual attributes when judging the visual appeal of a photograph, which was confirmed by the explanations of participants in this study. The participants’ explanation was based on their own visual appraisal because we did not provide them with any suggested criteria. In such circumstances, participants still managed to pick out those photographs of high and poor visual quality (Factor 1) and referred to a few aesthetic attributes to support their sortings. For example, participants could easily judge whether a photograph is sharp and colourful when explaining why they liked or disliked a given photograph.

### Interest-dependent preferences for different subjects

It is noteworthy that visual quality is not the only factor that determines the perceived attractiveness of a photograph. The influence of the subject is also significant. Here, the Factors 2, 3 and 4 (all related to subject) in factor analysis explained a total of 44% of the variance, which reflects how the subject of photographs affect the preferences of participants. Specifically, photographs of birds (Factor 2), iconic landscapes (Factor 3) and wetland-related subjects (Factor 2) were appreciated by participants. Preferences of visitors for subjects can be explained by their personal preferred local activities or nature attractions [25,50]. A study of tourists’ preferences for photographs of Kaikoura, New Zealand found that tourists’ interests in local natural attractions and activities significantly influenced their liked and disliked photographs: for example, ca. 22% of the participants were interested in maritime recreation, and they preferred the photographs of local maritime recreational activities [25]. Similarly, this study present that all the participants loaded on Factor 2 (Local Birds Encounter) are those interested in birds to some extent (having a specialised or general interest in birds, i.e. SB or GB).

Tourists showed their preferences not only for nature photographs of different types of subjects but also for the same type of subject (e.g. wildlife) with different detailed characteristics. In particular, Factor 2 addressed a specific subject: birds. This factor focused on the specific subject rather than aesthetic aspects, and included those participants who preferred to use the following criteria when evaluating the visual appeal of a photograph of the bird(s): whether the bird in the photograph is locally representative or morphologically impressive; if the behaviour is unique and interesting or if respondents had seen these creatures before. The importance of the morphological traits of the subject in wildlife (or plant) photographs was also supported by Lišková and Frynta [38], who suggested that people preferred birds with larger eyes, shorter necks and longer tails. In addition, Marešová, Landová [28] also reported that the perceived attractiveness of photographs of species of milk snake was related to the colouration of the subject (i.e. the snake). Hence, for science communicators within national parks, integrating the photographs of visually attractive natural attractions into interpretive materials is likely to be an effective means to enhance communication, because both high visual quality and having an attractive subject contribute to the perceived attractiveness of the photograph. Next, we present the specific implications of a perceived attractive photograph for interpretation of nature attractions.

It is interesting that the preferences of participants for subjects showed an interest-dependent pattern. For example, all the respondents loaded on Factor 2 were interested in birds, and most of them (five out of seven) were specialised bird enthusiasts (SB). This can be explained by Maple, Eagles [39], who suggested that birdwatchers mainly focused on birds and were only interested in the interpretive materials about birds when visiting national parks. For SB, their criteria for preferred photographs mainly included species, behaviour and ecology presented in the photographs, which can be supported by their knowledge and experience of birds and bird watching. It might reflect that SB tended to focus on the emotions expressed by the photograph more than objective attributes such as colourfulness. Also, those SB are knowledgeable about birds to some extent. They are thus able to tell whether the moment in the photograph is difficult to capture. For example, Participant 9 gave the following comments on Photo CK05 (Common Kingfisher with a fish): “*This is an amazing capture of a hunting kingfisher*. *Such a moment is very rare and very difficult to photograph*”, suggesting how an observer’s knowledge of birds helped him appreciate the photograph.

For those participants loaded on Factor 2 who have a general interest in birds (GB), morphological traits and familiarity of the birds in the photographs became important influencers. In addition, familiarity was another aspect participants took into account (for Factor 2). The findings in terms of familiarity match those of Axelsson [51], who explored the implications of a few psychological factors on the aesthetic appreciation of photographs, and suggested that familiarity was one of the major factors determining the perceived visual appeal of a photograph.

## Conclusion and implications

Photographs have already been considered as an important visual element in interpretive materials in natioan parks, as they are able to enhance visiting experience, communicate local natural stories and encourage future visitation [4,11,14]. Howver, the visual appeal of photographs is rarely considered by researchers in the above fields: the eixsting research in tourism focused more on what the photographs present and how this may attract visitors or potional visitors [11,13,14,27]. Our research filled the gap above—the visual aethetics is also an important aspect to be considered when selecting photographs for interpretation within national parks (e.g. signage, brounches and exhibitions in the visitor centre), and sometimes this is even more important then the subject of photographs (see the factors extracted). Photograph is a form of visual art, so they appraently have visual aesthetic values [52]. When such an visual art element is involved in interpretive materials or academic research, its characteristics as a form of art (i.e. aesthetics) should be considered rather than ignored. We confirmed a practical approach to evaluating the aesthetic value of photographs here: Acquine, because the evaluations and statements by participants showed that the photographs with high aesthetic scores were indeed aesthetically appealing. As aesthetics evaluation is highly cpmplicated and subjective when it is conducted by human observers [18], researchers, designders or marketers of national parks are encouraged to use Acquine or similar online automated methods to evaluate the aesthetics of photogrpahs. In addition, our reseearch also have methodolighcal contirbutions: in most existing studies using photograph-based Q method, the visual aesthetics of photgraphs are ignored, and participants’ specific interests in the subjects of phtographs are also seldm condisered when selenting photographs for the Q sets [24,25,27]. In the present study, we confirmed the importance of the above aspects. Future research using photograph-involved approach is encouraged to take the above factors into acoount.

It can be concluded that visitors within XNWP had varied preferences for photographs reflecting local nature stories, but they still shared some commonality in preferences when they assessed the perceived attractiveness of a photograph. Our findings also confirmed the “circle of representation” model by Jenkins [11] from a slightly different perspective: the photographs that preferred by tourists are also likely to be attractive to other tourists. We have successfully answered the qustion: what types of photographs of natural attractions are more attractive to the visitors. Specifically, participants’ appreciation was closely related to the visual qualities and subjects of the photographs. Those photographs with high visual qualities and attractive subjects were more successful in attracting attention. The visual quality of a photograph was determined by its aesthetic value (measured by Acquine); the attractiveness of the subject was closely related to participants’ interest in the subject, as well as the characteristics (visual, behavioural, ecological, etc.) of the subject. Our findings are able to guide the design of interpretive materials (particularly the selection of photographs) within national parks, and help to attract visitors with specific interests in different natural attractions by using photographs with different natual subjects.

## Supporting information

S1 Table The original data set. (XLSX)
